# Rewiring of IGF1 secretion and enhanced IGF1R signaling induced by co-chaperone carboxyl-terminus of Hsp70 interacting protein in adipose-derived stem cells provide augmented cardioprotection in aging-hypertensive rats

**DOI:** 10.18632/aging.205287

**Published:** 2023-12-11

**Authors:** Parthasarathi Barik, Wei-Wen Kuo, Chia-Hua Kuo, Dennis Jine-Yuan Hsieh, Cecilia Hsuan Day, Jayasimharayalu Daddam, Michael Yu-Chih Chen, V. Vijaya Padma, Marthandam Asokan Shibu, Chih-Yang Huang

**Affiliations:** 1 Graduate Institute of Basic Medical Science, China Medical University, Taichung, Taiwan; 2 Department of Biological Science and Technology, China Medical University, Taichung, Taiwan; 3 Laboratory of Exercise Biochemistry, University of Taipei, Taipei, Taiwan; 4 School of Medical Laboratory and Biotechnology, Chung Shan Medical University, Taichung, Taiwan; 5 Clinical Laboratory, Chung Shan Medical University Hospital, Taichung, Taiwan; 6 Department of Nursing, Mei Ho University, Pingtung, Taiwan; 7 Cardiovascular and Mitochondrial Related Disease Research Center, Hualien Tzu Chi Hospital, Buddhist Tzu Chi Medical Foundation, Hualien, Taiwan; 8 Department of Cardiology, Buddhist Tzu Chi General Hospital, Hualien, Taiwan; 9 Department of Biotechnology, Bharathiar University, Coimbatore, India; 10 Department of Medical Research, China Medical University Hospital, China Medical University, Taichung, Taiwan; 11 Department of Biotechnology, Asia University, Taichung, Taiwan; 12 Center of General Education, Buddhist Tzu Chi Medical Foundation, Tzu Chi University of Science and Technology, Hualien, Taiwan

**Keywords:** cardiovascular diseases, hypertension, aging, adipose-derived stem cells, insulin-like growth factor-1 (receptor) – IGF1(R), co-chaperone CHIP

## Abstract

Aging-associated cardiovascular diseases depend on the longitudinal deterioration of stem cell dynamics. The entire mechanism behind it is not completely understood. However, many studies suggest that endocrine pathways, particularly the insulin-like growth factor-1(IGF1) signaling pathway are involved in cardioprotection, especially in stem-cell treatments. Here, we investigated the role of a co-chaperone, carboxyl-terminus of Hsp70 interacting protein (CHIP) in the aspects of growth factor secretion and receptor stabilization in mesenchymal stem cells (MSCs). Briefly, we overexpressed CHIP in rat adipose-derived stem cells (rADSCs) and explored the consequences *in vitro*, and *in vivo*, in spontaneously hypertensive rats (SHR). Our data revealed that CHIP overexpression in rADSCs promoted the secretion of insulin-like growth factor-1 (IGF1) and IGF binding protein-3 (IGFBP3) as per immunoblot/cytokine array analysis. We also found that these results were dependent on the nuclear translocation of signal transducer and activator of transcription 3 (STAT3) in rADSCs. Further, the CHIP co-chaperone was also involved in the stabilization of the receptor of IGF1 (IGF1R); interactions between the beta transmembrane region of IGF1R, and the tetracopeptide repeat (TPR) domain of CHIP were evident. Importantly, after the transplantation of lentiviral CHIP overexpression of rADSCs (rADSCs^CHIP-WT^) into nine months aging-SHR led to an increase in their cardiac function - increased ejection fraction and fractional shortening (≈15% vs. control SHR) - as well as a decrease in their heart size and heart rate, respectively. Altogether, our results support the use of CHIP overexpressing stem cells for the mitigation of cardiac hypertrophy and remodeling associated with late-stage hypertension.

## INTRODUCTION

Pathological cardiac hypertrophy is a major cause of morbidity and mortality worldwide. Physiological hypertrophy may develop as an adaptive mechanism through increased blood pressure and heart volume overload during intense physical exercise. However, intrinsic cardiomyopathic stimuli or extrinsic stimuli for chronic conditions leads to pathological hypertension with increased risk of morbidity and sudden cardiac death, especially in left ventricular hypertrophy [1]. The renin-angiotensin-aldosterone system (RAAS) has been identified as one of the most important non-hemodynamic factors for the development of left ventricular hypertrophy [2]; particularly, high circulating levels of angiotensin II (Ang II) were involved in this process by the elevation of oxidative stress. Interestingly, in pathological stress conditions, oxidative stress can induce high vascular damage [3]. Importantly, the incidence of cardiovascular disease (CVD) increases in aged individuals [4]. In fact, aging affects cardiac function even in the absence of diseases. Hypertension in aged individuals, oxidative stress [5], and the release of ROS have a critical role in the involvement of growth arrest and the accumulation of misfolded proteins [6]. Reducing Insulin growth factor 1 (IGF1)-mediated signaling is directly related to aging and CVD [7]. Importantly, in the heart IGF1 signaling regulates various cellular mechanisms, including growth, metabolic homeostasis [8], and autophagy [9]. For instance, IGF1 ligand and its receptor, IGF1R interaction starts downstream signaling cascades in cardiomyocytes that eventually regulate their differentiation, proliferation, metabolism, hypertrophy [10], and protection from cell death.

MSCs have been known for the secretion of anti-inflammatory and angiogenic factors. Therefore, these cells should be considered for the development of cell-based therapeutics for regenerative medicine. A major problem in stem cell therapy is the limited amount of stem cells; however, tissues with higher-yielding capacity must be selected, such as the adipose tissue [11]. Most importantly, adipose-derived stem cell therapy is safe, immediately applicable for various diseases and also less invasive surgical measures to obtain the adipose tissue as compared to bone marrow tissue [12]. All around the world, the increasing rate of obese and overweight individuals has accelerated at alarming rates. Stem cells based-approach for a lifetime is to replace the mature adipocytes as it resides in adipose tissue. Importantly, the use of these cells in clinical medicine and different experimental injury models revealed major advantages, such as the abundance in nature, the easy isolation processes, and the lower rejection capacity [13]. Among the factors essential for the normal development and growth of stem cells, IGF1 is particularly important, however, abnormal IGF1 receptor (IGF1R)-mediated signaling was associated with the attenuation of stem cell survival [14]. Importantly, in aging, there is a decline in the expression of IGF1R [15]; consequently, the reduction of the IGF1R functions leads to various disorders as well as to many abnormalities. Co-chaperone CHIP, an E3 ubiquitin ligase, that interacts with HSP70 and further promotes the proteolysis mechanism [16]. Therefore, co-chaperone CHIP is an important player in the maintenance of stem cell growth, proliferation, and stemness. CHIP knockout mice model shows a decrease trend of lifespan and increment of aging phenotypes [17], that provides a connection between proteasome, co-chaperones and aging mechanism. The above players can potentially contribute to the attenuation of age-related cardiovascular diseases in stem cell-based therapies in aging.

However, chaperone-mediated growth factor secretion and receptor stabilization have not been fully explored. Therefore, here, we investigated the dual function of the co-chaperone CHIP, *in vitro*, in rADSCs, and *in vivo* using the aging-SHR model. We further investigated the role of CHIP in rADSCs-derived growth factor secretion (focusing particularly on IGF1 and IGFBP3), as well as stabilization of growth factor receptors, such as IGF1R and their cardioprotective effects after the transplantation in the aging-SHR rats.

## RESULTS

### Effects of co-chaperone CHIP overexpression on the IGF1 secretion in rADSCs

**Figure 1:**
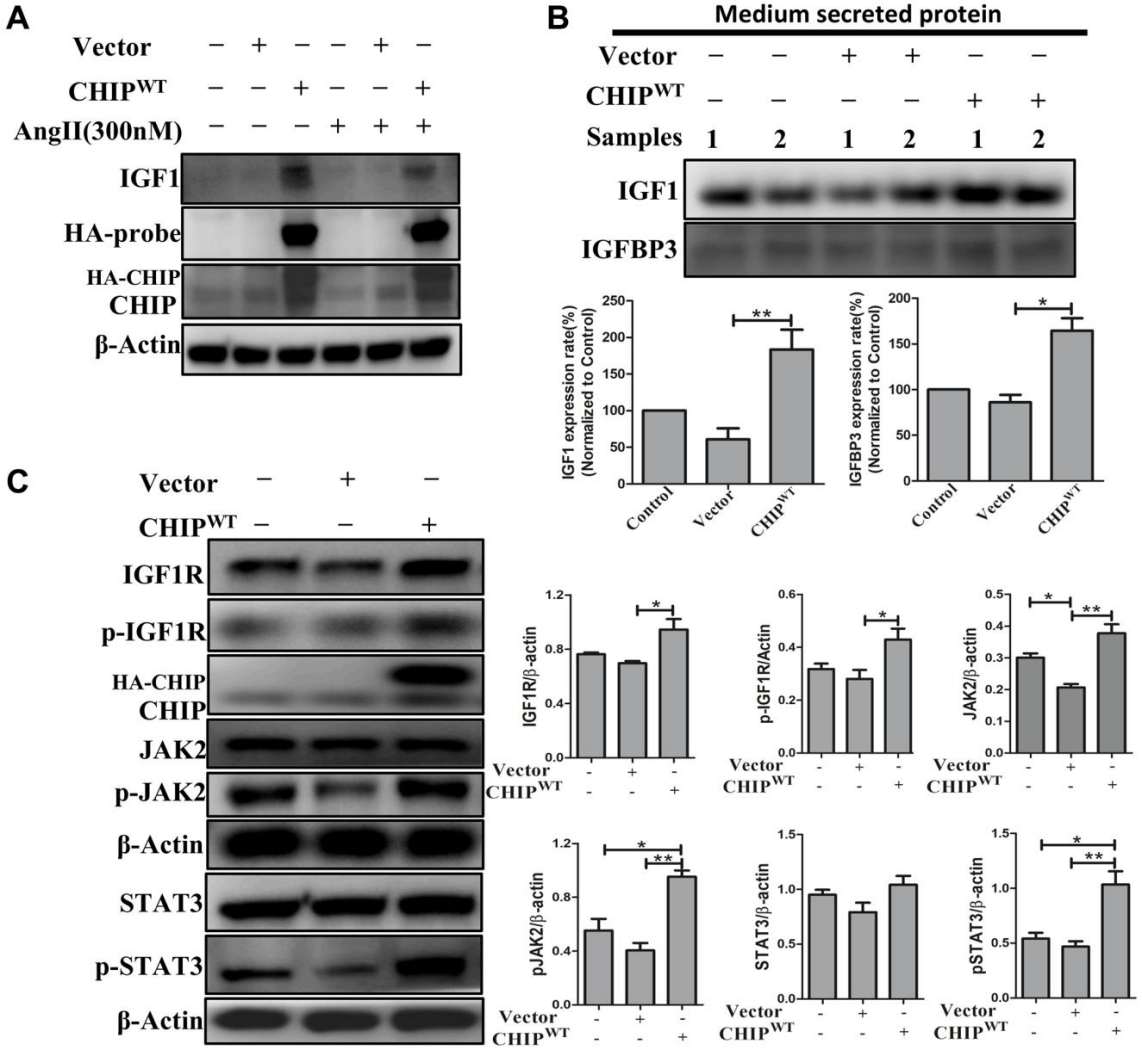
**Regulation of growth factors secretion (IGF1 & IGFBP3) in rADSCs.** (**A**) Effects of co-chaperone CHIP overexpression on IGF1 secretion against Ang II challenge for 24 h in rADSCs by immunoblot analysis. (**B**) Level of secreted proteins such as IGF1 and IGFBP3 was estimated by Western blot after 24 h transfection of HA-vector and HA-CHIP wild-type in rADSCs. (**C**) Expression levels of IGF1R and its downstream signaling proteins after overexpression with either HA-vector or HA-CHIP wild-type for 24 h were analyzed by Western blotting. (*N* = 3; ^*^p < 0.05; ^**^p < 0.01 indicate significant differences).

The co-chaperone CHIP is essential for the regulation of protein quality control and proteostasis effects in stem cells [18]. Interestingly, here we also report that the overexpression of the co-chaperone CHIP (CHIP^OE^) led to the secretion of IGF1 in rADSCs. Importantly, in adverse conditions, such as the Ang II challenge and the consequent increase in oxidative stress, the CHIP^OE^ positively regulated the expression of IGF1 by nullifying the effect of the Ang II challenge in rADSCs as per immunoblot analysis (Figure 1A). Intriguingly, IGFBP3 was reported to bind to IGF1 and the formation of a ternary complex [19]. Therefore, next, we evaluated the secretion of growth factors into the conditioned medium of rADSCs using a cytokine array. Surprisingly, in this study, the upregulation of IGF1 and IGFBP3 further confirmed that IGFBP3 binds to and stabilizes IGF1 in CHIP^OE^; of note, the secretion of IGF1 and IGFBP3 was higher CHIP^OE^-treated group (Supplementary Figure 1A). In this regard, these results were further confirmed by immunoblot analysis; a significant increase in the expression of IGF1 (^**^p < 0.01) and IGFBP3 (^*^p < 0.05) were observed in CHIP^OE^ stem cells (Figure 1C). In addition, we asked whether IGF1 could bind to its receptor and enhance downstream signaling in CHIP^OE^. The significant (^*^p < 0.05) upregulation of the expression of IGF1R in CHIP^OE^ rADSCs supported this hypothesis (Figure 1B). Importantly, the downstream active forms of Janus kinase (pJAK2) and the transcription factor STAT3 were also significantly increased in CHIP^OE^ rADSCs (^**^p < 0.01 vs. vector; Figure 1B). Moreover, CHIP^OE^ also regulated the activation of other downstream proteins such as pAKT (Supplementary Figure 1B). Taken together, our results suggest that the CHIP^OE^ in stem cells positively regulates the maintenance of cellular homeostasis and proteostasis mechanism to accelerate the secretion of growth factors such as IGF1 and IGFBP3, which further interacts with the IGF1R to promote the downstream survival pathway.

### Effects of CHIP overexpression on colocalization with IGF1Rβ in rADSCs

**Figure 2:**
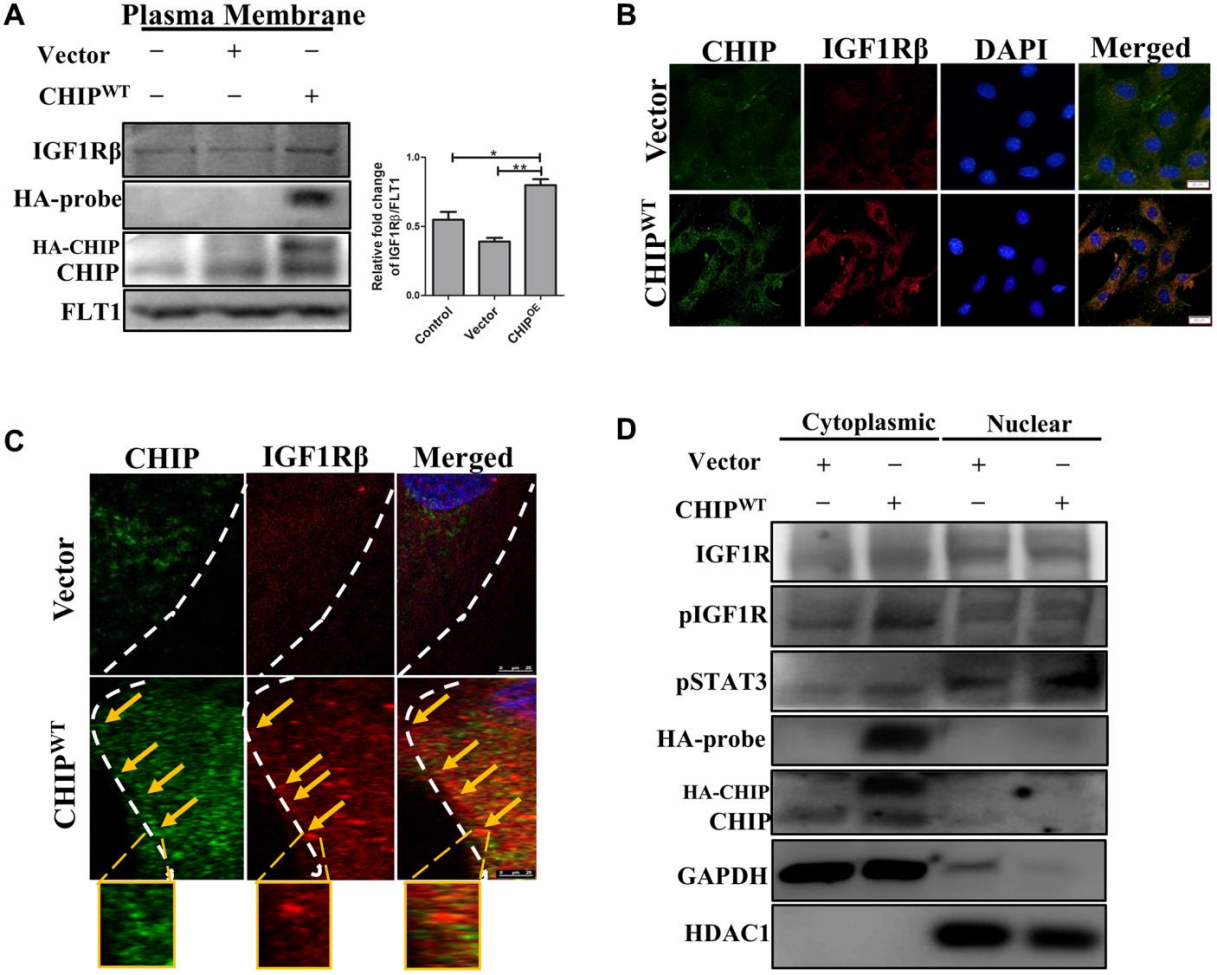
**Colocalization of IGF1R with co-chaperone CHIP in rADSCs.** (**A**) Level of IGF1Rβ in the membrane isolates was measured by Western blotting after overexpression of HA-vector/HA-CHIP wild-type for 24 h in rADSCs. (**B**) Cellular localization of CHIP and IGF1Rβ with or without CHIP overexpression in rADSCs. Scale bars = 20 μm. (**C**) Confocal microscope analysis to indicate the CHIP (green color-left) and IGF1Rβ (red color-middle) expression and colocalization (orange color-right) with or without CHIP overexpression in rADSCs. Scale bar = 10 μm. (**D**) Western blot analysis indicates that the cytoplasmic and nuclear extraction of various proteins expression, respectively. (*N* = 3; ^*^p < 0.05; ^**^p < 0.01 indicate significant differences).

It has been previously reported that ligand-receptor interactions promote the activation of the receptor tyrosine kinase domains of IGF1R, initiating downstream signaling [20]. Therefore, next, we explored the ligand-receptor interactions with a focus on the IGF1Rβ-subunits in CHIP^OE^ stem cells via membrane isolation. Importantly, immunoblot analysis revealed that a significant increase of IGF1Rβ expression in CHIP^OE^ vs. control and vector-transfected rADSCs (Figure 2A; ^**^p < 0.01 vs. vector, ^*^p < 0.05 vs. control). Furthermore, we measured the IGF1Rβ intensity by immunofluorescence analysis in stem cells, and the results aligned with the above ones (Figure 2B). Next, we asked whether the CHIP co-chaperone localizes together with the IGF1Rβ that is known to regulate the stabilization and maintain the expression of IGF1R. Intriguingly, confocal microscopy imaging indicated the occurrence of CHIP-IGF1Rβ interactions, as per the orange signals in CHIP^OE^ stem cells (Figure 2C). Overall, these data support the hypothesis that CHIP^OE^ in rADSCs enhances the expression and also stabilization by interacting with the IGF1R transmembrane (β) region. Hence, we found that the CHIP^OE^ regulated the activation of the STAT3 transcription factor, we then investigated its nuclear translocation by nuclear-cytoplasmic fraction analysis. Remarkably, the levels of pSTAT3 in the nuclei increased after CHIP^OE^ as compared to vector transfection in rADSCs (Figure 2D). Surprisingly, we also revealed the expression of IGF1R both in the cytoplasm and nuclear fractions, in line with a previous report on nuclear IGF1R [21]. Thus, our data strongly suggest that IGF1R (β-subunit) co-localizes with CHIP promoting the expression and activation of the transcription factor STAT3 and the consequent translocation into the nucleus in rADSCs.

### Disclosing the interaction between CHIP and the IGF1Rβ transmembrane region

**Figure 3:**
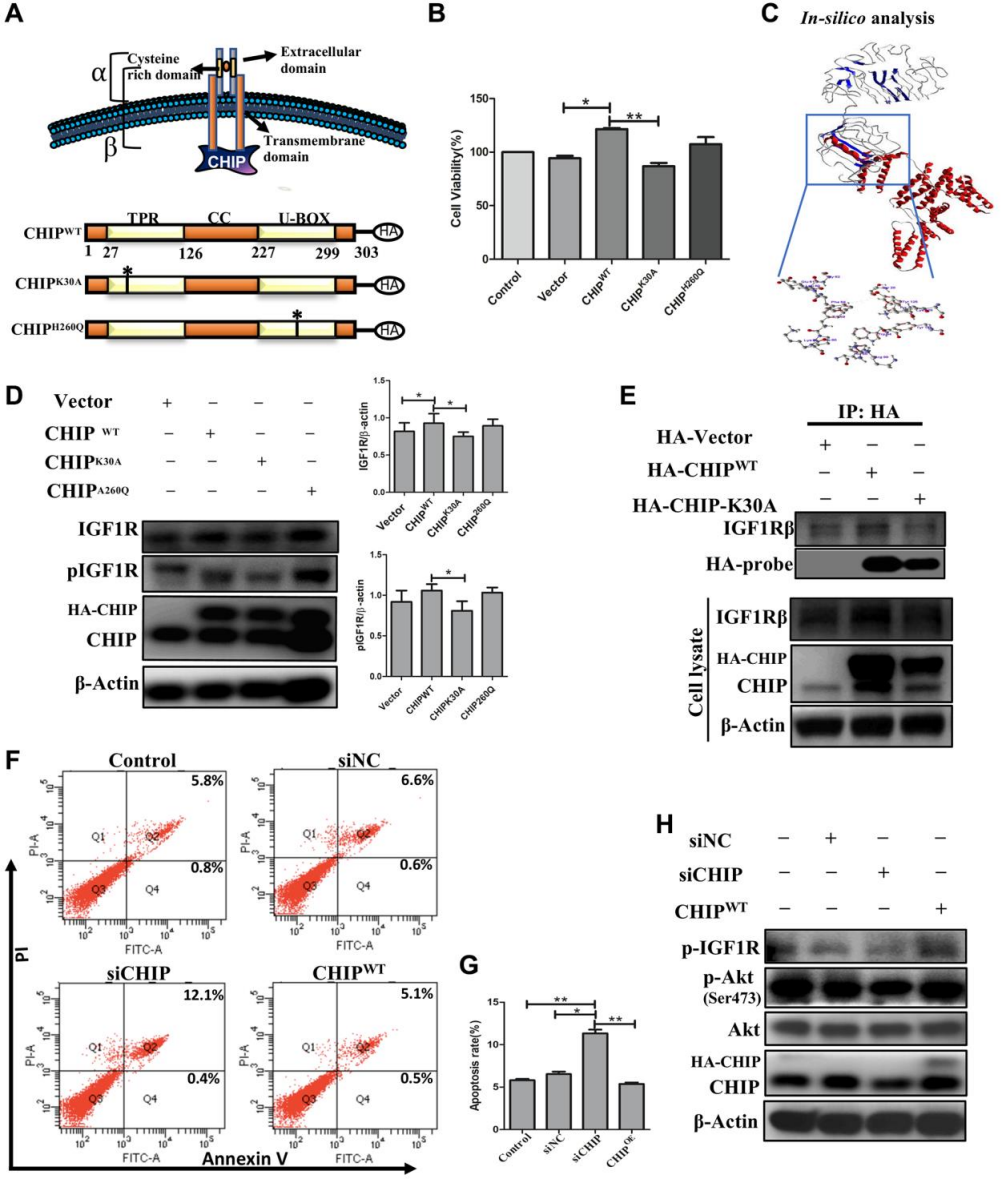
**Interaction of co-chaperone TPR domain of CHIP (CHIP-TPR) with IGF1Rβ.** (**A**) Schematic representation of CHIP and IGF1Rβ interaction. The lower figure indicates the CHIP wild-type and point mutation plasmids. (**B**) Cell viability rate was measured by MTT assay after transfection of HA-vector, HA-CHIP, and mutants of HA-CHIP (K30A, and H260Q) for 24 h. (**C**) The docking analysis showed the interaction between CHIP-TPR domain and IGF1Rβ. (**D**) rADSCs were transfected by HA-vector, HA-CHIP wild-type, and HA-CHIP two mutants (K30A, and H260Q) for 24 h and analyzed by Western blotting. (**E**) Co-immunoprecipitation (co-IP) analysis was performed by using rADSCs after transfection of HA-vector, HA-CHIP wild-type, and HA-CHIP-K30A for 24 h. (**F**, **G**) The apoptotic cells were analyzed by using a flow cytometer. (**H**) rADSCs were knocked down with siCHIP for 24 h and analyzed by Western blotting. (*N* = 3; ^*^p < 0.05; ^**^p < 0.01 indicate significant differences).

Since we observed a clear co-localization between CHIP and IGF1Rβ. Next, we analyzed the CHIP mutated forms and assessed the interaction with IGF1R; a schematic diagram illustrating the wild-type CHIP (CHIP-WT) protein and its proposed interaction with the IGF1Rβ whereas in bottom CHIP wild-type and its two mutants i.e., the N-terminal TPR (CHIP-K30A mutant) and the C-terminal U-box (CHIP-H260Q mutant) is represented (Figure 3A) and also ensured the size of wild-type and different mutants through enzymatic digestion (Supplementary Figure 1C). Importantly, the overexpression of CHIP-WT and CHIP-H260Q led to increasing cell viability, while the overexpression of CHIP-K30A did not show such effects in rADSCs by MTT assay (Figure 3B); importantly, these results were consistent with those in our previous report using H9c2 cells [22]. Further, the disclosure of the binding mechanism by *in silico* analysis, important domains were identified in CHIP and IGF1R as per the SBASE server (Supplementary Figure 1D). Remarkably, the binding results indicated the high interaction between the CHIP-TPR domain and the IGF1R transmembrane (β) domain (i.e., −154. 34 KJ/mol) (Figure 3C). Of note, the overexpression of the CHIP K30A-TPR mutant did not effectively upregulate the expression and activation of IGF1R; however, the overexpression of the CHIP-WT induced the expression and activation of IGF1R in rADSCs to a similar result found in CHIP-H260Q mutant form (Figure 3D). Further, to validate these results, we performed co-IP by using the anti-HA antibody in rADSCs cells. As expected, the co-IP results confirmed the interaction between CHIP wild-type and IGF1Rβ, while in the TPR mutant such interactions were lost (Figure 3E). In addition, another co-IP result confirmed the interaction between CHIP and IGF1R was remarkably high in CHIP^OE^ (Supplementary Figure 1E). Therefore, these results indicated that the interaction between these two proteins i.e., CHIP-TPR domain and the IGF1Rβ region. Previous results revealed that the depletion of CHIP reduces the stabilization of IGF1R [23], in line with our results showing that the CHIP^OE^ promotes the stabilization of IGF1R. Still, we decided to reproduce the results by CHIP knockdown, and additionally to understand its effects of apoptosis in rADSCs. Interestingly, we found knockdown of CHIP using small interference RNA (siRNA) in rADSCs led to an increase in the apoptosis rate significantly as per flow cytometry analysis (Figure 3F, 3G; ^**^p < 0.01 vs. CHIP^WT^ and control, ^*^p < 0.05 vs. vector). Moreover, CHIP knockdown prevented the activation and phosphorylation of IGF1R as well as of its downstream protein kinase AKT, as per immunoblot analysis (Figure 3H). Thus, overall, these results suggest that CHIP is important for cell survival via the promotion of IGF1R stabilization.

### Effects of the STAT3 knockdown on IGF1 expression in rADSCs

**Figure 4:**
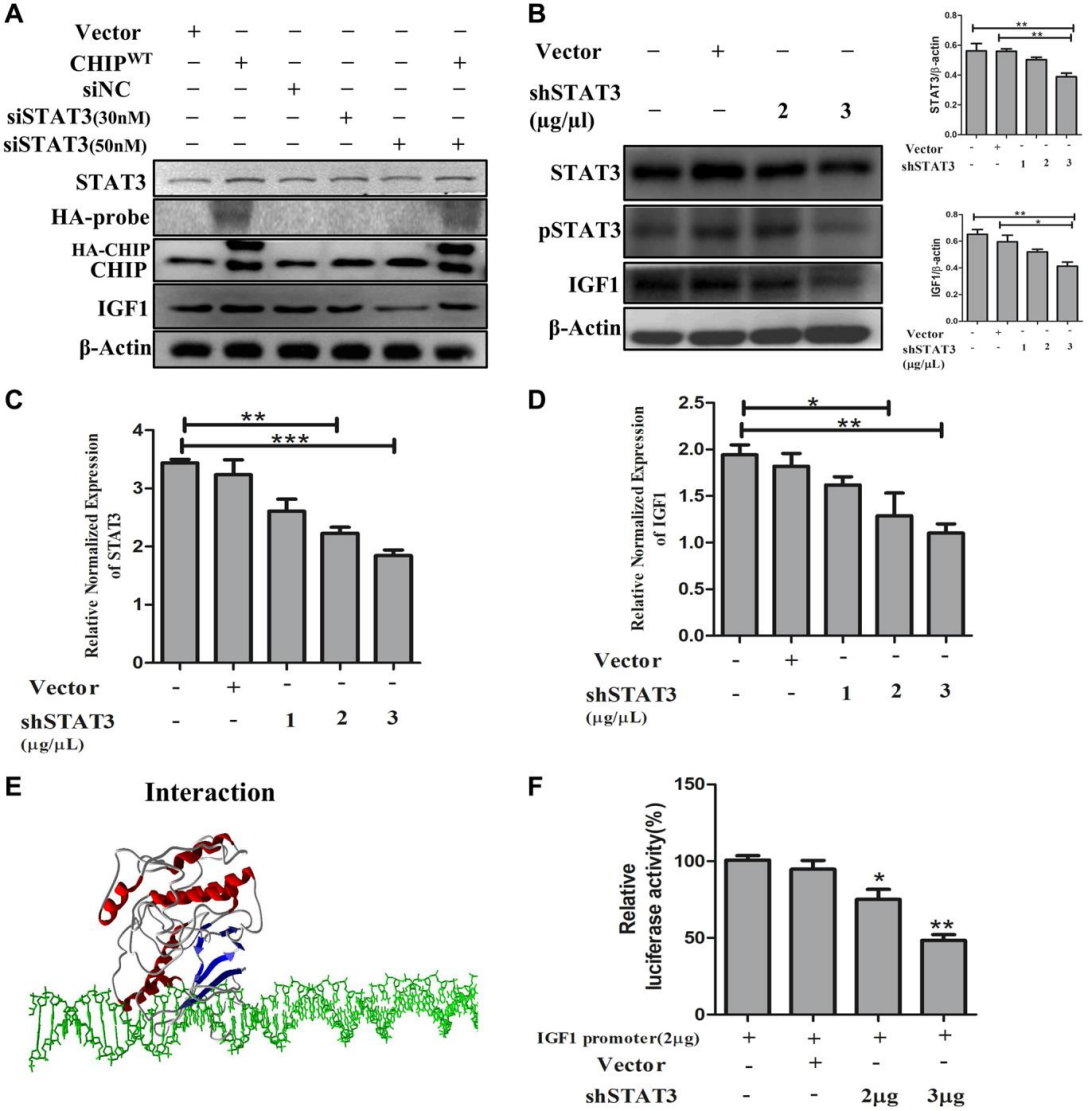
**STAT3 induces transcriptional activation of IGF1 in rADSCs.** (**A**) The dose-dependent inhibition of STAT3 for 24 h in rADSCs. (**B**) Knockdown of STAT3 by transfection of shSTAT3 for 24 h and its expression levels by immunoblot analysis. (**C**, **D**) The mRNA expression levels of STAT3 and IGF1 were determined by qRT PCR analysis. (**E**) *In silico* study illustrated the interaction between STAT3 and IGF1 promoter levels. (**F**) Transfection with PGL4-IGF1 promoter plasmid and silencing STAT3 expression with shSTAT3 for 24 h and further analysis by luciferase activity. (*N* = 3; ^*^p < 0.05; ^**^p < 0.01; ^***^p < 0.001 indicate significant differences).

The IGF1R is activated through STAT3-dependent IGF1 secretion in endocrine, paracrine, or autocrine fashions in stem cells [24]. Therefore, next, we performed STAT3 knockdown in rADSCs. Importantly, our results showed that STAT3 knockdown prevented the expression of IGF1 expression in a dose-dependent way. Interestingly, CHIP^OE^ after STAT3 knockdown promoted the activation of STAT3 by abrogating the effect of siSTAT3 with an increase in the expression of IGF1 in rADSCs (Figure 4A). Furthermore, rADSCs treated with shSTAT3 showed a dose-dependent reduction of the expression of both STAT3 (^*^p < 0.05 vs. control and vector) and IGF1 (^*^p < 0.05 vs. control and vector) as per Western blot analysis (Figure 4B). Notably, we have analyzed the mRNA levels by qRT PCR, as expected the data revealed that the higher dose of shSTAT3 regulates the significant reduction of STAT3 and also IGF1 expression in rADSCs (Figure 4C, 4D). Interestingly, a previous report showed that the regulation of the secretion of IGF1 by STAT3 is dependent on an interaction with the IGF1 promoter region [25]. Therefore, next, we performed molecular docking *in silico* analysis of STAT3 and the IGF1 promoter using the PDB structures. Importantly, docking analysis showed an excellent binding efficiency with a high energy score (i.e., −125.46 KJ/mol) between STAT3 and the IGF1 promoter region (Figure 4E). Furthermore, we validated these results using a luciferase-based promoter assay in rADSCs. Importantly, the resulting luciferase activity indicated that the IGF1 promoter activity was significantly reduced in shSTAT3 transfected vs. vector-transfected and control cells (Figure 4F). Altogether, these results suggest that the IGF1-promoter activity is regulated by STAT3, the key player behind the effect due to CHIP^OE^ in rADSCs.

### Effects of CHIP-overexpressing stem cells on Ang II-Induced hypertrophy in H9c2 cells

**Figure 5:**
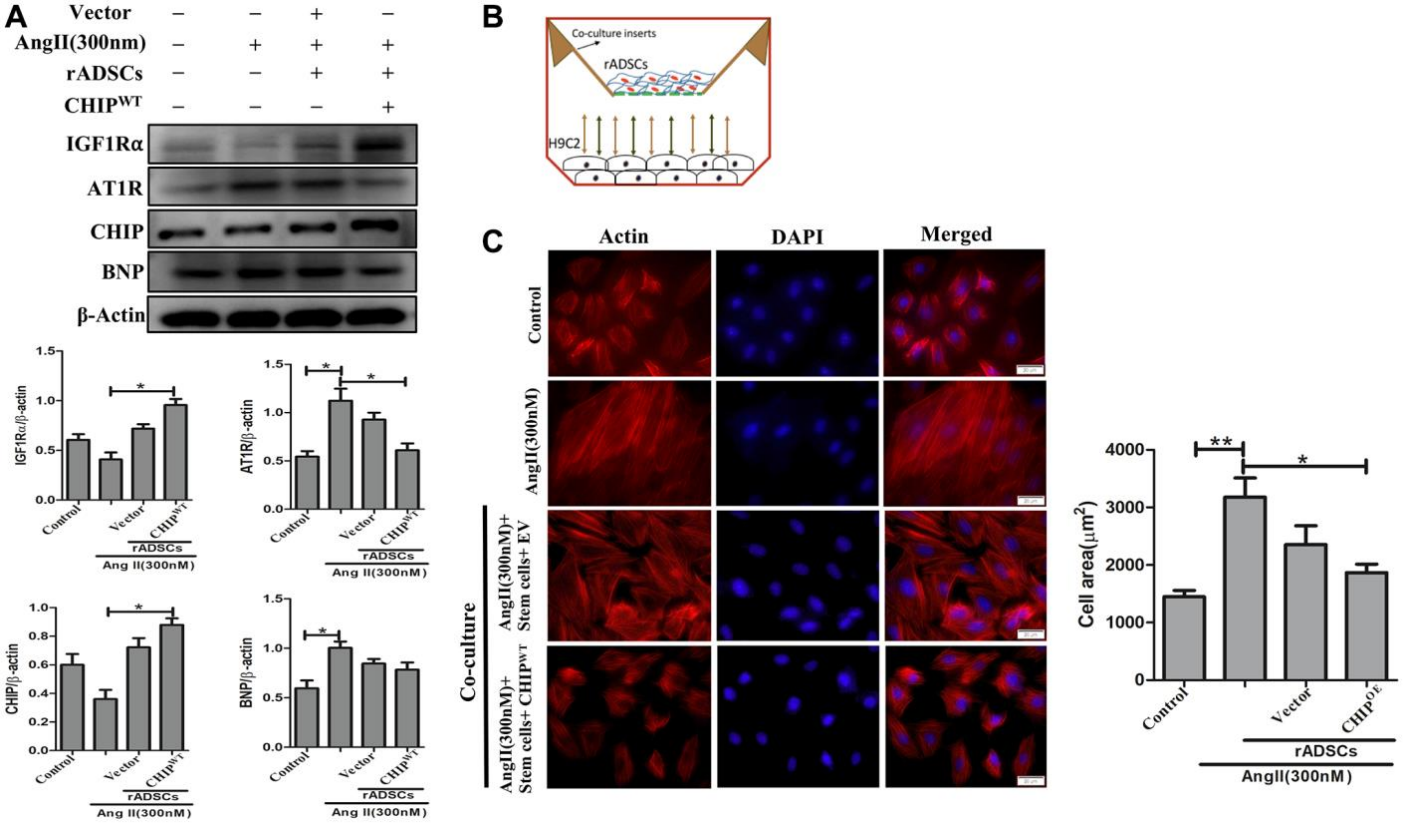
**CHIP-overexpressing ADSCs attenuate Ang II-induced hypertrophy in H9c2 cells.** (**A**) The co-culture analysis with rADSCs and H9c2 to determine the expression levels of survival and hypertrophic markers in H9c2 cells. (**B**) The schematic diagram of the co-culture technique showing rADSCs in the upper chamber and H9c2 cells in the bottom chamber. (**C**) Rhodamine-phalloidin staining measures the area of H9c2 cells after being challenged with Ang II for 24 h. Scale bar = 20 μm. (*N* = 3; ^*^p < 0.05; ^**^p < 0.01 indicate significant differences).

Mesenchymal stem cells have the property to secret angiogenic and anti-inflammatory trophic factors [26]. To mimic the SHR model, we induced hypertrophy in H9c2 cells *in vitro* by using Ang II challenge then co-cultured these cells with CHIP^OE^ rADSCs to disclose the potential cardioprotective effects of CHIP co-chaperone. Importantly, the immunoblot results confirmed the significant upregulation of the extracellular domain of IGF1R (IGF1Rα) as well as of the co-chaperone CHIP (^*^p < 0.05); however, a significant downregulation of the expression of hypertrophic markers such as Ang II receptor type 1 (AT1R) and brain natriuretic peptide (BNP) were observed in Ang II-treated H9c2 cells (Figure 5A; ^*^p < 0.05). In addition, rhodamine-phalloidin staining results revealed that the treatment with CHIP^OE^ stem cells significantly reduced the hypertrophic effect (^*^p < 0.05) against Ang II in H9c2 cells compared to control and stem cells alone treatment (Figure 5B, 5C). Moreover, CHIP^OE^ stem cells regulate secretomes such as IGF1 and IGFBP3 that regulate paracrine signaling to abolish the hypertrophic effect against the Ang II-treated H9c2 cells.

### Regulation of cardiac function after administration of CHIP-overexpressing rADSCs

**Figure 6:**
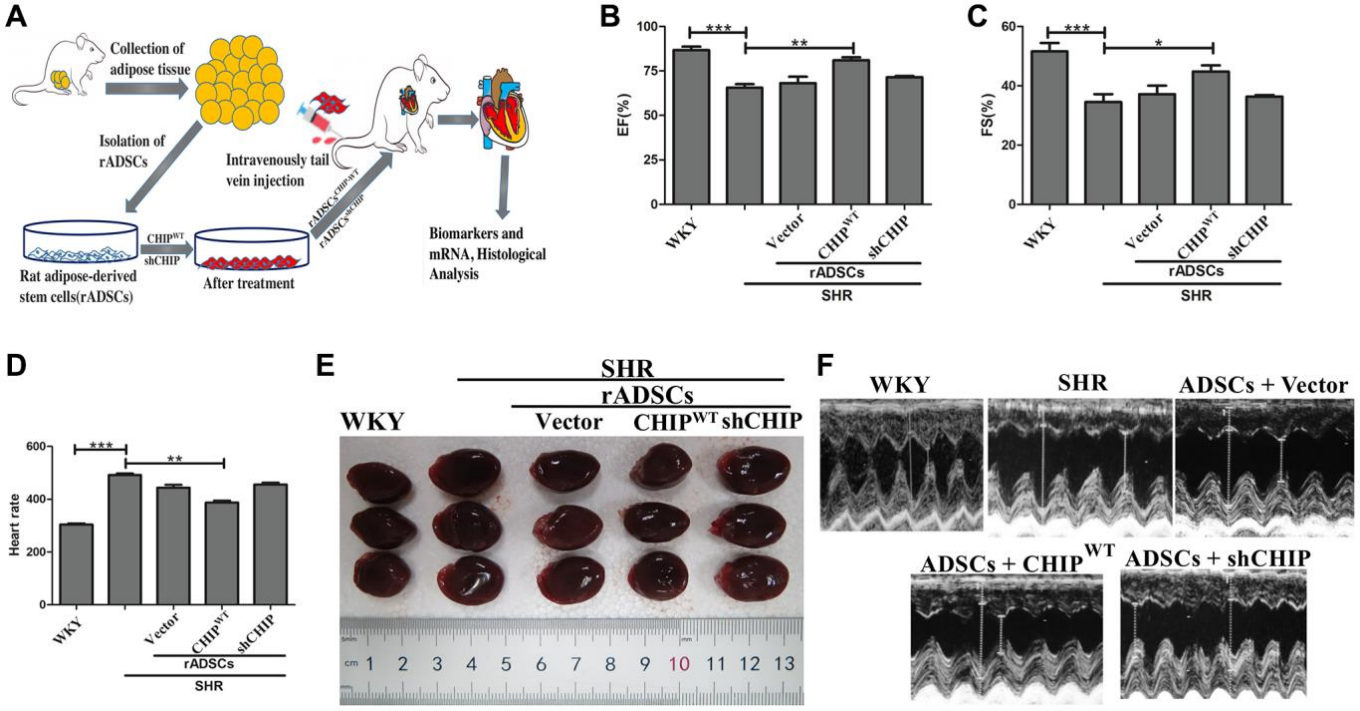
**Transplantation of CHIP-overexpressing ADSCs (rADSC^CHIP-WT^) regulates cardiac function in the aging-SHR model.** (**A**) The schematic diagram illustrates the method for ADSC isolation and their transplantation into aging-SHR. (**B**, **C**) EF (%) and %FS were measured through echocardiographic analysis on WKYs, SHRs, and stem cell treated SHRs (*N* = 3). (**D**) Heart rate was measured by tail-cuff method of different experimental groups (*N* = 7). (**E**) Morphological assessments of rat hearts from different experimental groups, (*N* = 3). (**F**) The LVIDd and LVIDs obtained by echocardiographic analysis of different experimental groups, respectively. (^*^p < 0.05; ^**^p < 0.01; ^***^p < 0.001 indicate significant differences).

Furthermore, we sought to investigate the effects of CHIP^OE^ rADSCs *in vivo*; we used a tail vein injection-mediated autologous transplantation approach. First, we ensured the complete model, from the isolation of ADSCs from the adipose tissues to the engineered cells and finally to their intravenous injection into aging-SHR was illustrated (Figure 6A). Then, we experimented by using nine months of WKY and SHR rats which showed higher variation in their heart rate (Supplementary Figure 2A). Further, we analyzed their cardiac function using echocardiographic measurements after the transplantation of different rADSCs into the SHR after the transduction. Surprisingly, the transplantation of CHIP overexpressing stem cells (rADSCs^CHIP-WT^) into SHR rats led to a significant increase in the ejection fraction (EF, ≈15%) and the fractional shortening (FS, ≈15%), as compared to those in the other groups of SHR rats (Figure 6B, 6C; ^**^p < 0.01). Additionally, the transplantation of rADSCs^CHIP-WT^ into SHR attenuated significantly their heart rate (^**^p < 0.01) (Figure 6D). We also checked this parameter before the stem cell administration to ensure the difference between before and after the treatment (Supplementary Figure 1D). Next, we focused on morphological changes. Importantly, the hearts of SHR-rADSCs^CHIP-WT^ and SHR-rADSC^shCHIP^ showed a clear morphological difference (Figure 6E). In fact, the blood pressure overload in SHR-rADSC^shCHIP^ and in control SHR promoted cardiac hypertrophy as per the higher heart size and weight, remarkably, the hypertrophic response was smaller in SHR-rADSCs^CHIP-WT^ (Figure 6F, Table 1). Moreover, SHR-rADSCs^CHIP-WT^ showed significantly lower LVIDd and LVIDs vs. those in SHR and SHR-rADSC^shCHIP^ (Figure 6G). In summary, the results suggest that SHR-rADSCs^CHIP-WT^ promoted cardioprotection via the regulation of the heart rate, systolic, diastolic pressure, and also hypertrophy *in vivo* conditions.

**Table 1:** Echocardiographic and cardiac morphological assessment of cardiac function.

	**WKY (*n* = 7)**	**SHR group (*n* = 7)**	**rADSCs**		
			**Vector**	**CHIP^WT^**	**shCHIP**
LVIDd (mm)	6.9 ± 0.23	8.67 ± 0.22^##^	7.94 ± 0.26	7.42 ± 0.24^**^	8.48 ± 0.28
LVIDs (mm)	3.36 ± 0.11	5.52 ± 0.13^##^	4.1 ± 0.16	3.5 ± 0.12^*^	5.35 ± 0.23
Body weight (g)	369.73 ± 4.15	360.54 ± 5.19	363.91 ± 4.38	365.21 ± 5.47	357.57 ± 6.32
Tibia length (mm)	41.08 ± 0.61	40.81 ± 0.41	40.65 ± 0.54	40.84 ± 0.46	40.88 ± 0.53
WHW (mg)	1233.4 ± 17.05	1391.08 ± 16.62^#^	1370.75 ± 17.80	1325.48 ± 18.36^*^	1384.62 ± 15.38^#^
LVHW (mg)	972.85 ± 12.67	1123.17 ± 18.82^##^	1109.42 ± 18.39	1047.92 ± 10.11^*^	1114.87 ± 17.78^#^
WHW/Tibia length (mg/mm)	30.28 ± 0.65	34.10 ± 0.60^#^	33.75 ± 0.63	31.40 ± 0.51^*^	33.89 ± 0.45^#^

Abbreviations: LVIDd: Left ventricular internal diameter end-diastole; LVIDs: Left ventricular internal diameter end-systole; WHW: Whole heart weight; LVHW: Left ventricular heart weight. Values are presented as mean ± SEM. ^#^*p* < 0.05, ^##^*p* < 0.01, compared to WKY group; ^*^p < 0.05, ^**^p < 0.01 compared to SHR group.

### Effects of transplanted CHIP-overexpressing ADSCs on IGF1R signaling cascade in aging-SHR model

**Figure 7:**
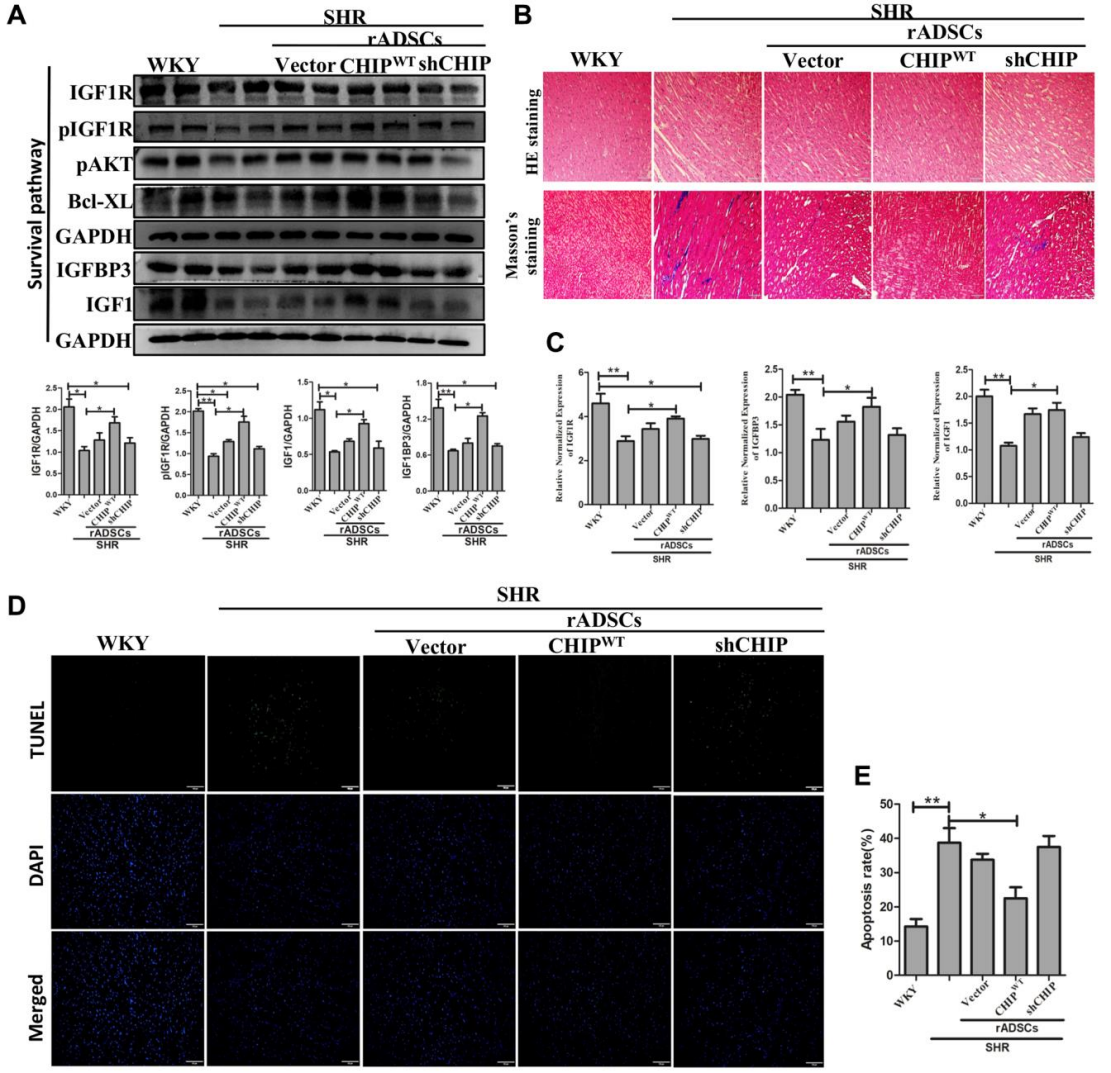
**CHIP-overexpressing ADSCs (rADSC^CHIP-WT^) in the aging-SHR model inhibit cardiac remodeling.** (**A**) The immunoblotting detects the survival markers expression of heart tissue in different experimental groups after the transplantation. (**B**) Hematoxylin-eosin (HE) staining (upper panel) and Masson's trichrome staining (lower panel) were performed to evaluate tissue arrangement and collagen accumulation in different experimental groups. Scale bar = 100 μm. (**C**) Heart tissue mRNA expression levels of IGF1R, IGFBP3, and IGF1 in WKY, SHR, and SHR-treated groups were analyzed by qRT PCR analysis. (**D**, **E**) TUNEL assay to detect the apoptosis rate of different experimental groups. (*N* = 3; ^*^p < 0.05; ^**^p < 0.01 indicate significant differences).

Although CHIP knockout mice showed a decline in life span has been reported [17], the role of the CHIP overexpressed stem cells is still unknown in the aging-SHR rat model. Moreover, the immunoblot analysis revealed a higher expression of IGF1R and pIGF1R as well as of downstream proteins in SHR-rADSC^CHIP-WT^; importantly, the expression levels of IGFBP3 (^*^p < 0.05) and IGF1 (^*^p < 0.05) were also significantly higher in SHR-rADSC^CHIP-WT^ group (Figure 7A). Moreover, Hematoxylin-eosin (HE) staining revealed larger interstitial spaces between tissues due to the loss and degeneration of cardiomyocytes in SHR vs. both WKY rats and SHR-rADSC^CHIP-WT^, the same was not observed in SHR-rADSC^shCHIP^ and SHR-rADSC^vector^. In line with these results, Masson’s trichrome staining clearly emphasized the high amount of collagen deposition with the blue color formation and fibrosis occurrence in the hearts of SHR and other treated groups as compared to those of WKY and SHR-rADSC^CHIP-WT^ (Figure 7B). Furthermore, immunohistochemistry results confirmed the higher expression levels of IGF1R in WKY and SHR-rADSC^CHIP-WT^ groups vs. those in SHR, SHR-rADSC^shCHIP^, and SHR-rADSC^vector^ groups (Supplementary Figure 2B). Next, we looked at the mRNA expression levels in heart tissues using qRT PCR analysis. Importantly, the expression of important survival markers such as IGF1R, IGFBP3, and IGF1 was significantly increased in the heart tissues of WKY and SHR-rADSC^CHIP-WT^ vs. SHR and SHR-rADSC^shCHIP^ groups (Figure 7C). Remarkably, the results of the TUNEL assay indicated a significant higher rate of TUNEL^+^ cells in SHR and SHR-rADSC^shCHIP^ and SHR-rADSC^vector^ as compared to that in WKY rats and SHR-rADSC^CHIP-WT^ groups; these results clearly showed that the CHIP-overexpressing rADSCs administration protects from apoptosis in the heart tissues of aging-SHR rats (Figure 7D, 7E). Taken together, the result confirms that administration of SHR-rADSC^CHIP-WT^ regulates the cardiac function by upregulating the IGF1R signaling cascades and prevents the apoptosis in the aging-hypertensive conditions.

## DISCUSSION

Our results demonstrate that the CHIP co-chaperone induces the secretion of IGF1 and stabilizes IGF1R via the interaction between the CHIP-TPR domain and the IGF1R (β) region in rADSCs. Furthermore, we have shown CHIP promotes the improvement of the cardiac function of SHR in aging conditions. Remarkably, a clear correlation between the molecular co-chaperone CHIP and the mammalian lifespan *in vivo* was defined; probably this is due to the absence of CHIP and the consequent decrease in IGF1 signaling lead to pre-mature aging through the overload of damaged proteins in the cells [27]. Spontaneously developing hypertension in aging is a fatal disorder accompanied by the increased thickening of cardiac walls, the permanent loss of cardiomyocytes, the occurrence of fibrosis, and the decline of metabolic functions [28]; this results in cardiac remodeling [29].

**Figure 8:**
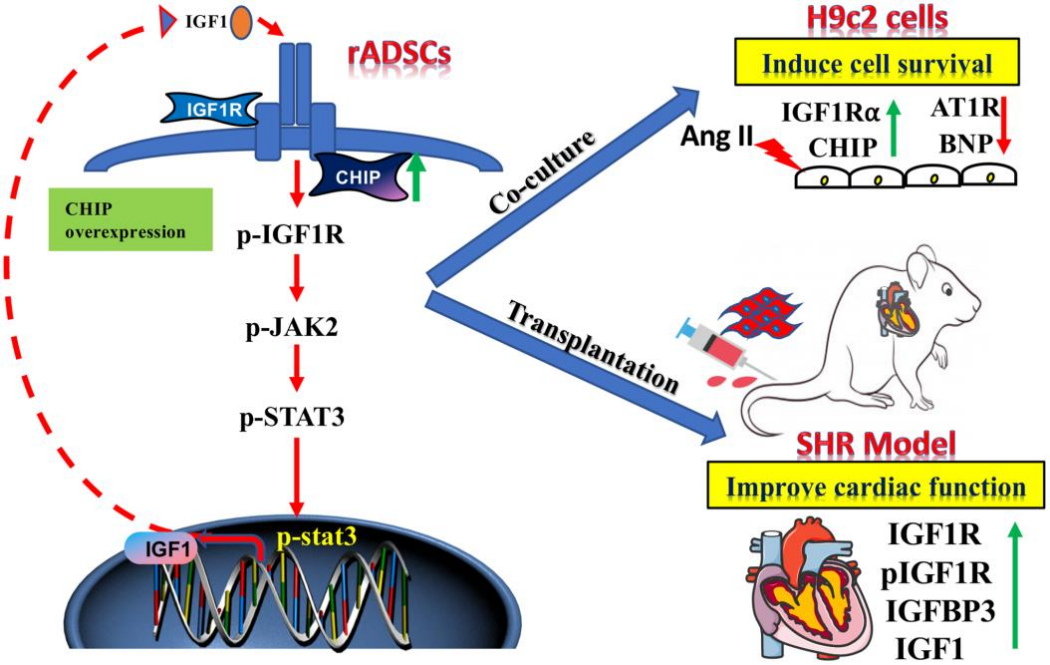
**The schematic diagram demonstrating the association of elevated levels of co-chaperone CHIP with better therapeutic benefits of rADSCs.** Co-chaperone CHIP overexpression on rADSCs regulates the IGF1 secretion. Furthermore, transplantation of engineered rADSCs in aging-SHR model augments the cardioprotective effects and suppresses cardiac remodeling.

Clinical trials continue to demonstrate that ADSCs are a good option to treat heart failure secondary to hypertension as they can reduce the cost of cell therapies. Stem cell-based heart-targeted therapies and regenerative medicine strategies should act synergistically to achieve the true cardiac repair. Among stem cells, ADSCs have for a long time been considered as a promising candidate for stem cell-based therapies due to the secretion of beneficial growth factors such as IGF1, VEGF, and HGF that promote cell survival, neovascularization, and angiogenesis [30]. However, in aging conditions perivascular adipose tissue-derived stromal cells regulate vascular remodeling [31]; so, maintaining the cellular proteostasis in the aging condition is a more challenging and important approach. In fact, in support of this hypothesis, here we show that CHIP^OE^ in stem cells secreting high levels of IGF1 prevented the development of hypertrophy against Ang II in H9c2 cells both *in vitro* and *in vivo* study (Figure 8). The use of Ang II *in vitro* mimics of our hypertension rat model as Ang II is involved in cardiac hypertrophy [32]. Additionally, rADSCs can differentiate into various kinds of cells with their tremendous potential in regenerative medicine. Importantly, here, we observed that the transplantation of rADSCs^CHIP-WT^ led to the improvement of the cardiac function of SHR, supporting the application of MSCs for cardiac function. This effect was dependent on the expression of CHIP as the transplantation of rADSC^vector^ and rADSC^shCHIP^ did not improve the cardiac function of SHR. Therefore, the beneficial effects of stem cells are dependent on the secretion of cytokines and growth factors (e.g., IGF1) [33]. From our cell model, we have presumed that the secretion of paracrine factors provides a better environment for the function of the heart in the aging-associated SHR.

CHIP is a major cytoplasmic chaperone responsible for the regulation of various proteins through either their stabilization or their degradation. Importantly, here we show that CHIP interacts with IGF1Rβ through the TPR domain promoting its stabilization and consequently the activation of downstream protein targets. MSCs are already known to secrete potent anti-fibrotic factors including matrix metalloproteinases 9, 14, and 2; they inhibit cardiac fibroblasts and attenuate fibrosis [34]. Interestingly, in this study, the heart tissues of SHR-rADSCs^CHIP-WT^ showed remarkably lower fibrosis levels as compared to those of SHR, SHR-rADSC^vector^, and SHR-rADSC^shCHIP^ groups as per the Masson’s trichome staining. Therefore, we cannot exclude the hypothesis that CHIP overexpression in rADSCs may impact the expression of metalloproteinases, which in turn prevent the development of fibrosis and collagen deposition. Here, we show that under stress conditions such as the challenge with Ang II *in vitro* or aging-related hypertension *in vivo*, chaperones play a major role in the regulation of protein folding-refolding and protein stability; our *in vitro* and *in vivo* results in CHIP overexpression support this hypothesis.

It is noteworthy that, CHIP overexpression regulates the expression of IGF1R, promoting the survival of rADSCs. Importantly, a previous report from our lab demonstrated that CHIP stabilizes HSF1 also through its TPR-domain [22]. Here, in this study, we show that the overexpression of CHIP increases the stability of IGF1R via interactions between the CHIP-TPR domain and IGF1Rβ. We believe that the overexpression of CHIP triggers the IGF1 secretion through the pIGF1R-pJAK2-pSTAT3 pathway to regulate the transcriptional activity in rADSCs. In fact, STAT3 was reported to act as a transcription factor for IGF1 secretion in MSCs [35]. Importantly, it is hypothesized that the survival and proliferative capacity of MSCs can be enhanced by the maintenance of a certain stem cell microenvironment [36]. As abovementioned, secreted factors such as placental growth factor, VEGF, and IGF are essential to cell survival. Notably, transplantation of rADSCs^CHIP-WT^ in the SHR model we observed an increased expression of IGF1 and IGFBP3 and further supporting the importance of CHIP overexpressing stem cell-based therapies in aging and hypertension; therefore, insulin/IGF1 plays an important role in development and metabolism throughout life, from ontogeny to advanced age.

## CONCLUSION

In summary, we designed a strategy for the induction of IGF1 signaling via the overexpression of the CHIP co-chaperone in stem cells for the treatment of aging hypertension conditions. Importantly, we have shown a promising effect *in vivo*, associated with the increased secretion of IGF1 and IGFBP3 as well as also with the increased stabilization of IGF1R. We believe this strategy can be used as a future treatment for aging-related hypertension and cardiovascular damages. Moreover, since the CHIP^OE^ provides a tractable means to improve stem cell survival and proliferation, this strategy must also be considered in regenerative medicine in general, for the treatment of various pathological conditions. Lastly, our results provide insight into the molecular mechanisms behind the regulation of cardiac remodeling and the augmentation of cardiac function.

## MATERIALS AND METHODS

### Isolation and characterization of adipose-derived stem cells (ADSCs)

The isolation and characterization of rat adipose-derived stem cells (rADSCs) were described and characterized in our previous report [37]. Briefly, ADSCs were isolated from rat epididymal fat tissues using type 2 collagenase (col-II) (0.2% in PBS). rADSCs were then cultured in low-glucose Dulbecco’s Modified Eagle’s Medium (DMEM; Invitrogen, Carlsbad, CA, USA) supplemented with 10% FBS (Invitrogen), 100 U/ml penicillin (Invitrogen), 100 mg/ml streptomycin (Invitrogen) and 2 mM L-glutamine (Invitrogen) at 37°C with 5% CO_2_.

### Western blot analysis

This Western blot was performed and described in our previous report [38]. Briefly, the protein was extracted from lysis buffer: 50 mM Tris, pH 7.5, 0.5 M NaCl, 1.0 mM EDTA, pH 7.5, 10% glycerol, 1 mM BME, 1% IGEPAL-630, and a proteinase inhibitor cocktail (Roche Molecular Biochemicals, Penzberg, Germany), and concentrations were determined using the Bradford method (Bio-Rad, Hercules, CA, USA). The proteins were separated by 8–12% SDS-PAGE and transferred onto PVDF membranes (Millipore, Belford, MA, USA). Membranes were blocked with 5% blocking buffer and blotted with different specific primary antibodies (listed in Supplementary Table 1) at 4°C overnight. Immunoreactivity bands were detected using the Alphamager 2200 digital imaging system (Digital Imaging System, Commerce, CA, USA). All of the secondary antibodies (anti-rabbit, mouse and goat, HRP-conjugated) were purchased from Santa Cruz Biotechnology (Santa Cruz, CA, USA). All reagents were purchased from Sigma-Aldrich (St. Louis, MO, USA).

### Co-immunoprecipitation (Co-IP)

Co-IP in rADSC lysates was performed using the protein G magnetic bead system (Millipore, Belford, MA, USA) as per the manufacturer’s instructions. Briefly, after the cells were harvested and lysed in, 20 mM Tris-HCl (pH 7.5), 1% NP-40, 150 mM NaCl, and 1 mM EDTA, 400 μg of protein were incubated with an anti-CHIP antibody (listed in Supplementary Table 1) overnight at 4°C after the different treatment. Afterward, the immunoprecipitated proteins were eluted at 95°C for 8 minutes and analyzed by SDS-PAGE.

### Plasmids, shRNAs, and gene transfer

The plasmid encoding the CHIP protein was gifted by Dr. Jeng-Fan Lo (Yang-Ming Medical University, Taipei, Taiwan) [22]. Plasmids were transfected using the PureFection™ Nanotechnology-based transfection reagent (System Biosciences, CA, USA) following the manufacturer’s protocol. Moreover, three other plasmids, including pMD.G, pCMVΔR8.91, and shCHIP were purchased from National RNAi Core (Academia Sinica, Taipei, Taiwan) and used in this study; they were co-transfected together with CHIP (wild type)-encoding lentiviruses from Sinobiosis (Cat# RG83573-ACGLN, Beijing, China) into 293T kidney cells using polybrene (Sigma-Aldrich, St. Louis, MO, USA). Thereafter, the culture medium was supplemented with puromycin and viable cells were collected and used for treatment.

### Promoter assay

This assay was performed as described in our previous report [39]. Briefly, the IGF1 promoter-luciferase construct (PGL4.1/IGF-RE; Promega, Madison, WI, USA) was co-transfected with different shRNAs into rADSCs using the jetPRIME^®^ reagent (Polyplus-transfection, Illkirch-Graffenstaden, France) according to the manufacturer’s protocol. Luciferase activity was then detected by using the luciferase kit (Promega, Madison, WI, USA).

### Cellular fractionation

Subcellular protein fractions of rADSCs (nuclear, cytosol, and membrane) were obtained using the “nuclear, cytosol, and membrane fractionation kit” (BioVision, Milpitas, CA, USA) according to the manufacturer’s protocol. The isolated fractions were then quantified using the Bradford assay (Bio-Rad, Hercules, CA, USA) and subjected to Western blot analysis.

### Confocal microscopy

The rADSCs were cultured chamber slides and then fixed with 4% paraformaldehyde for 20 min, and permeabilized (0.1% Triton X-100 in 0.1% sodium citrate) for 10 min at room temperature. Afterward, cells were rinsed with PBS, blocked with 5% goat serum, and further incubated with specific primary antibodies overnight (listed in Supplementary Table 1). Thereafter, cells were washed and incubated with Alexa Fluor^®^ 546 goat anti-rabbit IgG secondary antibody (Invitrogen, Carlsbad, CA, USA); nuclei were counter-stained using DAPI (Sigma-Aldrich, St. Louis, MO, USA). Finally, the expression levels of CHIP and IGF1Rβ were analyzed using a Leica SP2 confocal spectral microscope (Leica, Wetzlar, Germany).

### Co-culture of rADSCs and H9c2 cells

The following method was described in our previous report [37]. Briefly, H9c2 cells and rADSCs were co-cultured by using Millicell Hanging Cell Culture Insert (6-well PET 0.4 μm, Millipore, Merck KGaA, Darmstadt, Germany) and maintained in a 5% CO_2_ humidified incubator. Of note, H9c2 cells were seeded into the lower chamber in the presence of Ang II for 24 h. Then rADSCs (contained 1 × 10^5^ cells/well) were seeded into the upper Transwell chamber and were placed with H9c2 cells in 6-well culture dishes for 24 hours after transfected with CHIP wild-type and vector plasmid, respectively.

### Animal experiments

All animal experiments were performed in accordance with the Guidelines for the Care and Use of Laboratory Animals (National Institutes of Health, Publication No. 85-23, revised in 1996); all protocols were approved by the Animal Research Committee of Hualien Tzu Chi Hospital, Buddhist Tzu Chi Medical Foundation, Taiwan (108-IACUC-71). Eight-weeks of male Wistar Kyoto rats (WKY) and Spontaneously Hypertensive rats (SHR) were purchased from BioLasco Taiwan Co., Ltd., (Taipei, Taiwan). After nine months, animals were segregated into five groups (*n* = 7 per group): WKY, SHR, SHR injected with stem cells (1 × 10^7^ cells/rat) transduced with the lentiviral (LV)-vector (SHR-rADSCs^vector^), LV-CHIP^WT^ (SHR-rADSCs^CHIP-WT^), and LV-shCHIP (SHR-rADSCs^shCHIP^), for 24 h; animals were followed-up for four weeks. Rats were provided with standard laboratory food and water *ad libitum* and maintained under 12 h light-dark cycles at 24 ± 2°C. After the treatment period, the animals were weighed, anesthetized with isoflurane, and sacrificed by decapitation. The hearts were then collected and stored at −80°C.

### RNA extraction and qRT PCR analysis

Total RNA from the cells and left ventricular heart tissues was extracted and purified using the Quick-RNA™ MiniPrep kit (Zymo Research, Irvine, CA, USA) according to the manufacturer’s instructions. Afterward, RNAs purity was determined and cDNAs were obtained using the iScript™ cDNA synthesis kit (Bio-Rad, Hercules, CA, USA). Then, qRT PCR was then performed using specific forward and reverse primers (listed in Supplementary Table 2) and the SYBR Green PCR Master Mix (Bio-Rad) as per the manufacturer’s instructions; the CFX96™ Quantitative Real-Time system was used. All measurements were performed in triplicate; 40 cycles of amplification were performed. Gene expression was normalized to that of *U6*; the threshold cycle (Ct) value was determined for the different genes using the 2^ΔCt^ method, as per the following equation:


ΔCt=Ct microRNA−Ct U6 rRNA

### Echocardiography and tail-cuff method

Echocardiography was performed by using a sector transducer (5 to 8 MHz) or a linear transducer (12 MHz) (Vivid 3, General Electric Medical Systems Ultrasound, Haifa, Israel) according to the manufacturer’s instructions. Briefly, under isoflurane anesthesia, measurements were made in the M-mode and 2D images were obtained after the observation of at least six cardiac cycles. Moreover, a sphygmomanometer (BP-2010; Softron, Tokyo, Japan) was used to detect the heart rate of 10 weeks and 10 months (WKY and SHR) rats before and after the treatment via the tail-cuff method [40]. All of the cardiac measurements were made at room temperature (RT); measurements were repeated three times and the average was considered for further representation in the bar diagram.

### Flow cytometry analysis

The following method was described in our previous report [41]. Briefly, the treated cells were collected by trypsinization after 24 h and washed with 1X PBS three times then stained with Annexin V-FITC and PI staining (BD-Biosciences, Franklin Lakes, NJ, USA) as per the manufacturer’s instructions. The apoptosis analysis was performed by flow cytometry at the FACS Core Facility, China Medical University, Taiwan, using a FACS Canto™ system (BD FACScanto, BD-Biosciences, Franklin Lakes, NJ, USA). Further, the cells were gated to obtain the singlets after those cells in each quadrant of the fluorescein isothiocyanate (FITC-A) versus PI plot. At least 10,000 events were acquired by flow cytometry.

### Statistical analysis

All experiments were performed at least three times, independently; data are represented as the mean ± standard error of the mean (SEM). The *P*-value of *p* < 0.05 was considered statistically significant. Statistical analyses were performed by using the unpaired student’s *t*-test (comparison between two groups) and the one-way ANOVA test followed by *post hoc* Tukey’s Honestly Significant Difference tests (multiple comparisons); the GraphPad Prism 5 statistical software was used (GraphPad Inc., San Diego, CA, USA).

## Supplementary Materials

Supplementary Figures

Supplementary Tables
