# Supplementary material for: Rewiring of IGF1 secretion and enhanced IGF1R signaling induced by co-chaperone carboxyl-terminus of Hsp70 interacting protein in adipose-derived stem cells provide augmented cardioprotection in aging-hypertensive rats
Source: Aging (Albany NY). 2023 Dec 11;15(23):14019–38. doi: 10.18632/aging.205287 (PMC10756089; doi:10.18632/aging.205287)
Supplement: Supplementary Figures [file aging-15-205287-s001.pdf]

SUPPLEMENTARY FIGURES

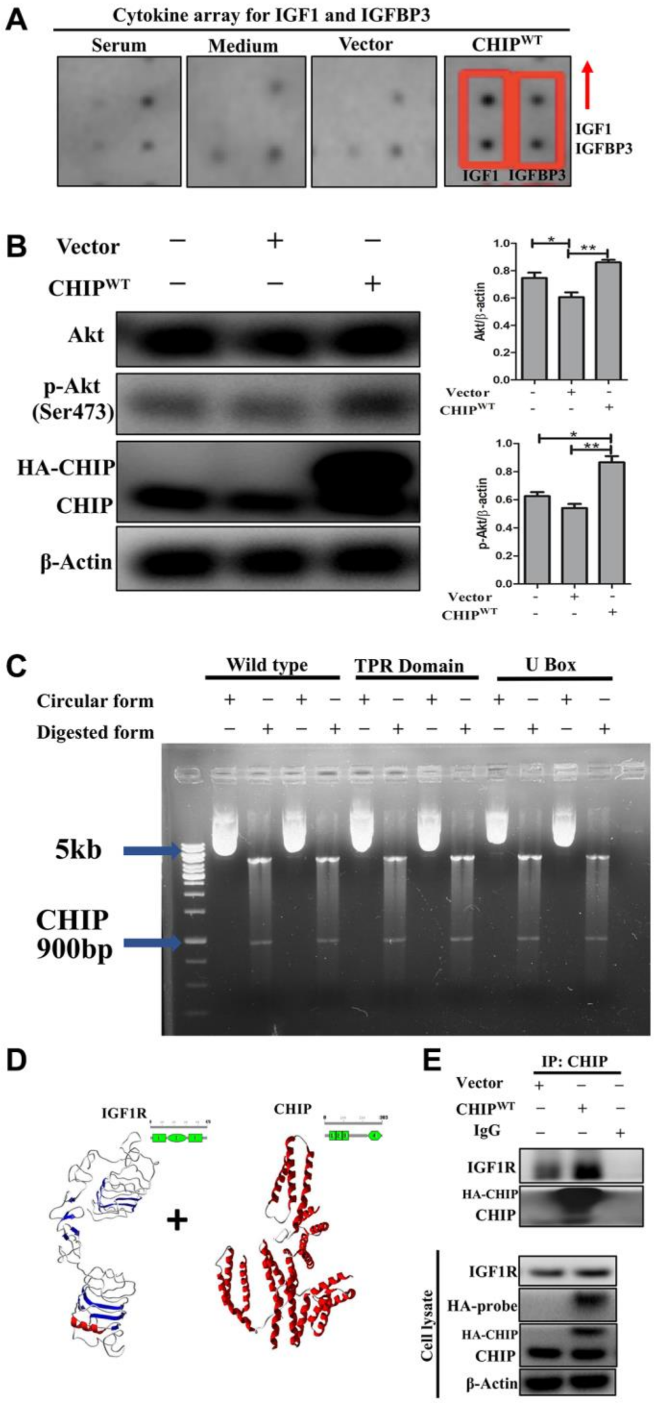

Supplementary Figure 1. Effects of CHIP overexpression on IGF1 secretion, size confirmation of wild-type and mutant forms, the PDB structure of IGF1R and CHIP co-chaperone. (**A**) Cytokine array to determine the secretion level of IGF1 and IGFBP3 in the medium of different treatment groups. (**B**) The immunoblot data indicated AKT and pAKT expression levels after overexpression of HA-vector/HA-CHIP wild-type for 24 h in rADSCs. Values represent as mean ± SEM (N = 3; $^{*}p < 0.05$; $^{**}p < 0.01$ indicate significant differences). (**C**) The agarose gel electrophoresis data showed the CHIP plasmid enzymatic digestion of wild-type and two mutant forms. (**D**) The PDB structures of IGF1R and CHIP protein. (**E**) Co-IP was done by transfection of HA-vector/HA-CHIP wild-type for 24 h and measured by immunoblotting.

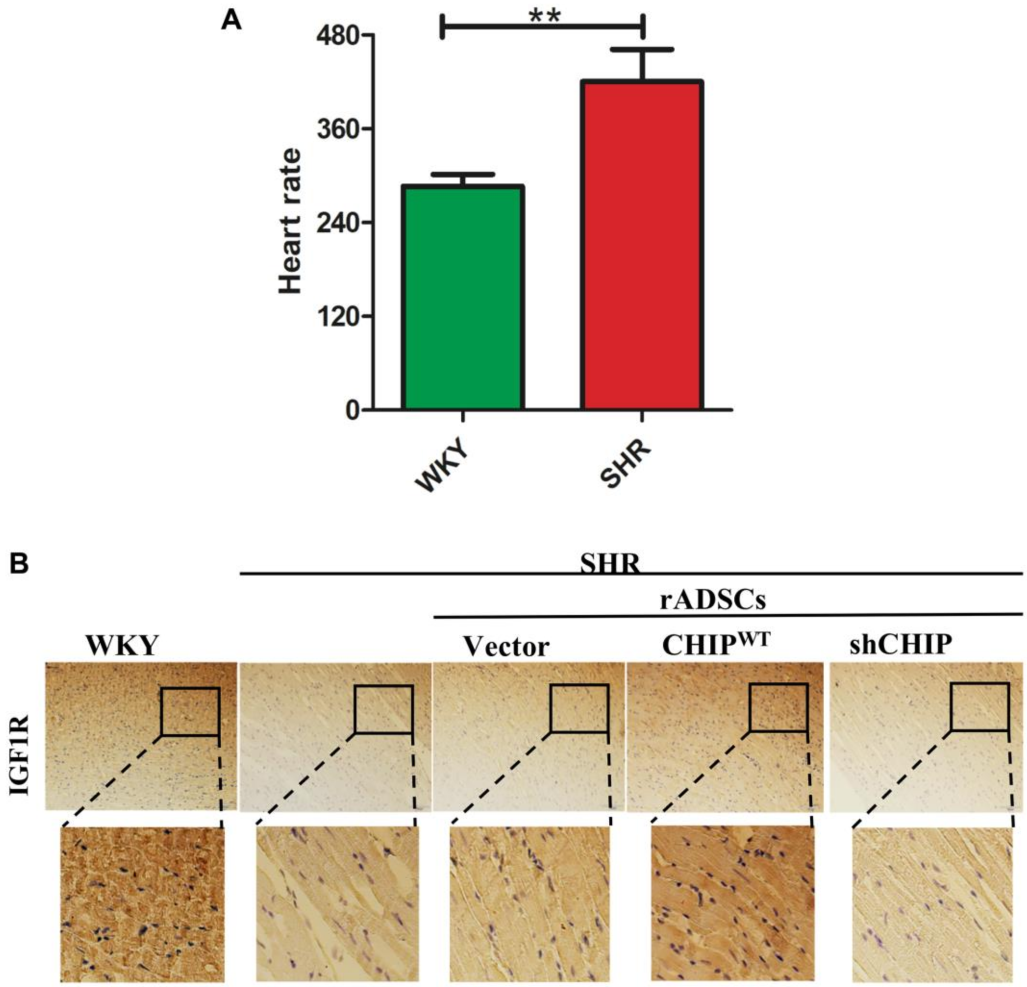

Supplementary Figure 2. Heart rate analysis and IGF1R immunohistochemistry of WKY, SHR and SHR-treated groups. (**A**) Heart rate analysis was performed by tail-cuff method for the WKY and SHR rats before treatment. Values represent as mean ± SEM ($N = 7$; $^{*}p < 0.05$; $^{**}p < 0.01$ indicate significant differences). (**B**) Immunohistochemistry for IGF1R expression of different experimental groups.
