# Supplementary material for: Rewiring of IGF1 secretion and enhanced IGF1R signaling induced by co-chaperone carboxyl-terminus of Hsp70 interacting protein in adipose-derived stem cells provide augmented cardioprotection in aging-hypertensive rats
Source: Aging (Albany NY). 2023 Dec 11;15(23):14019–38. doi: 10.18632/aging.205287 (PMC10756089; doi:10.18632/aging.205287)
Supplement: Supplementary Tables [file aging-15-205287-s002.pdf]

SUPPLEMENTARY TABLES

Supplementary Table 1. Antibodies list.

Antibody name	Provider	Cat.log number
IGF-1	Abcam	ab36532
CHIP	Santa Cruz	sc-66830
IGFBP3 (H-98)	Santa Cruz	sc-9028
IGF-IRβ (F-1)	Santa Cruz	sc-390130
Flt-1 (H-225)	Santa Cruz	sc-9029
HA-probe (F-7)	Santa Cruz	sc-7392
IGF-IR (7G11)	Santa Cruz	sc-81464
p-IGF1R (Tyr1161)	Santa Cruz	sc-101703
JAK2	Cell Signaling	#3230
p-JAK2 (Tyr1007/Tyr1008)	Cell Signaling	#3771
Stat3 (K-15)	Santa Cruz	sc-483
p-Stat3 (Tyr705)	Cell Signaling	#9145
HDAC1 (c-19)	Santa Cruz	sc-6298
GAPDH (6C5)	Santa Cruz	sc-32233
p-Akt1/2/3 (Ser473)	Santa Cruz	sc-7985
Akt1 (B-1)	Santa Cruz	sc-5298
AT1R (Angiotensin II type 1 receptor)	Abcam	ab18801
IGF-1Rα (H-78)	Santa Cruz	sc-7952
BNP	Bioss	bs-2207R

Supplementary Table 2. Primers sequence.

No.	ID name	Sequences
1	Forward – IGF1R	AAAACCATCGATTCTGTGACG
	Reverse – IGF1R	GGTTCTTCAGGAAGGACAAGG
2	Forward – IGFBP3	GGATCCTGAGCCTCTCATGC
	Reverse – IGFBP3	GGTGTCTGTCCTTGAGGGTG
3	Forward –IGF1	CCTCCTCGCATCTCTTCTACCT
	Reverse – IGF1	CTGCTGGAGCCATACCCTGTG
4	Forward – STAT3	GACATCAGTGGCAAGACCCA
	Reverse – STAT3	GGCGCTTGTCCAACAACAAA
5	Forward-Xhol-IGF1-Oilgo	AAACTCGAG TCTCGGAG
	Reverse-HindIII-IGF1-Oilgo	AAAAAGCTT GTGTAGGC
